# A Remarkable Case of Double Monstrosity in an Adult

**Published:** 1866-01

**Authors:** 


### MISCELLANEOUS.

### *A Remarkable Case of Double Monstrosity in an Adult.*

The following is an account of a very remarkable monstrosity in an adult who has lately arrived in this country. He presented himself at our office, and we have requested Mr. Ernest Hart, of St. Mary's Hospital, to carefully examine the parts. He reports as follows:

Jean Battista dos Santos, native of Faro in Portugal, is nineteen years of age, about five feet seven inches in height, well nourished, and symmetrically formed, with the important exception of the singular monstrosity here to be described. He is bronzed in tint, of intelligent expression, and well featured. When dressed in ordinary costume, he has all the activity and general appearance of any other well grown lad, concealing with complete success the supernumerary appendages which he bears.

He possesses two complete and well-formed penes, placed side by side, and a large central third leg and foot. Each penis is in itself complete in its general anatomical characters. They are placed laterally, each about an inch from the median line of the pubes. They are of more than average dimensions, each measuring $4\frac{3}{4}$ while pendant. The prepuce is retracted. The latter is $4\frac{1}{4}$ inches in circumference ; the right somewhat smaller, $3\frac{2}{3}$ inches. When the bladder acts, it expels its contents through both penes at the same moment. Under excitement, both become simultaneously erect; and other functions are performed by the two simultaneously. On the outer side of each is attached a fully-developed scrotum and testis. Between them hangs a shrunken scrotum, which contained two testes until he was ten years old, when, as he says, they ascended into the abdomen. He refuses to have bougies passed, so that it cannot be ascertained where the urethral passages communicate, and how they pass into the bladder, or whether this is normal in structure. He describes himself as possessing considerable virile power. It is difficult to say how the crura of the penes are attached internally, and on this subject it might be premature to hazard an opinion.

The third limb is of complex character, and presents some remarkable peculiarities. It exhibits marked evidences of the abortive effect at the production of twins, which is the key to the study of this singular monstrosity. At the first sight it seems to consist roughly of a large thigh, with an abortive leg, dislocated and bent up in front of it, and a misshapen foot, also dislocated.

Thus, to begin with the foot, it will be seen that it is really a coalescence of two feet, more or less perfect. The central toe is a consolidation of two great toes ; on the right are the four toes of that foot, tolerably perfect ; on the left are four toes, irregular in development, the second dwarfed to a tubercle, the third clubbed and bifid, the fourth and fifth tolerably regular. The tarsus and the metatarsus are fairly developed in double ; and on each border may be seen the projecting parts of the fifth metatarsal bones. The projections at the junction of the foot with the leg, which might fairly be mistaken each for an astragalus, are, in fact, the double projecting ends of a bifid tibia. On examining the plantar aspect of the foot, a double heel is very clearly made out. It is remarkable that the terminal part of the foot is devoid of ordinary sensation ; but he does not complain of its ever feeling cold or causing him pain. The foot is dislocated. The breadth of the foot at its terminal extremity is 7 in. ; in the middle $6\frac{1}{2}$ in. ; its length on the dorsum 4 in. ; its length on the sole 5 inches.

The leg is, as already stated, dislocated and fixed anteriorly on the thigh ; while its extremities project over the dorsum of the foot. The fibula could not be made out. The tibia is *bifurcated* at its lower extremity, and terminates in two prominate masses with distinct though rounded malleoli. The skin is all partially sensitive. The only movement which can be given to other parts of the limb is that of partial extention, soon checked both by its displacement and by the fold of skin which connects it with the anterior aspect of what represents the thigh. The length of the leg is $7\frac{1}{2}$ in.; around the lower expanded and bifurcated end it measures 9 in.; at the part where the tibia bifurcates 5 inches.

The thigh, or what represents it, is fleshy and somewhat pyramidal in shape; seen from the front, and in its usual position, it looks much like an ordinary thigh centrally situated between the two legs, and running upwards and backwards. It is freely movable. On being more nearly examined, it is seen to be attached to a third bone. This articulates, by a joint having very free motion, to the arch of the pubes. Seen posteriorly, it is observed to terminate above and behind by a thick clubbed projecting extremity of which the greater part is free and projects backward. The skin over the upper free part of the back of the bone is marked by a deep depression or puckered cul-de-sac, upwards of an inch deep, of which the significance is open to various interpretations. The thigh measures 9 in. round below, and 17 in. above, at its thickest part. Here it is flattened from side to side; and there is a considerable mass of what is probably fat and muscular tissue. There is, however, no power of voluntary movement in this or in any part of the limb.

The bone by which the direct attachment to the pelvis is effected is very short—about three inches in length. Its existence may easily be overlooked, for in front the skin passes continuously upwards from the thigh over it without any fold or marking. Its presence or mode of attachment are determined by grasping the limb firmly by its perineal attachment, and moving the thigh upon this upper piece. There is so much skin, fat, and cellular tissue around this short bony piece, that it is difficult to determine its precisive shape; but it will be seen to be irregularly curved and concave backwards. At its pubic attachment it moves freely; below the limb moves on it by a hinge-joint. From the free movement at the pubic articulation, the limb can at will be twisted round and made to project posteriorly, so that it is not all seen in front, and does not project there.

What is the pathological significance of this bone? During life it would be difficult to decide with certainty. It is not probably a pelvic bone, misshapen and centrally attached—the osteal congener of the true penis? On examining the perineum posteriorly it will be observed that there is a tendency in the skin running down over the central limb to assume the characters of a double perineum. The true anal aperture is deviated by about an inch from the median line; and the puckered cul-de-sac in the skin over the clubbed and enlarged upper posterior extremity of the thigh is suggestive of an abortive second anus. Then is this mass of bone only the upper end of a femur, with the trochanters excessively developed? or does it contain other abortive osteal elements?

There are suggestions which cannot probably be converted into certainties of one sort or the other during the life of the young man. On the other hand, he remembers to have been told that when he was a year old the third limb projected more stiffly than it does now, and a Portuguese chemist, officiating as surgeon, broke the limb at some part, so as to make it less cumbrous. He believes that it was at the juncture of the lower leg to the thigh that this was affected, and that the leg was then bent upward and forward in the position in which it now is. Examination renders this probable, since it is certainly dislocated into an unnatural position, and has only a false joint. But it is possible that at this time the neck of the thigh-bone was broken away from the body, and that the upper bone is that neck remaining attached to the arch of the pubes by a ball-and-socket joint, and making a false joint with the body of the bone.

However this may be, there is ample evidence from the examination of the whole limb to show that it is an abortive effort at a double extremity, which explains the presence of a double penis by the suggestion of an attempt at twins. So that this is, in fact, a case of a man having attached to him the lower limbs and penis of a partly-developed twin. What is particularly remarkable is the perfectly co-equal development of the two penes and testes, and their completely equal adaptation to the general functions of the individual organism of their bearer. It is true that the right penis is somewhat smaller in circumference than the left, but he states that they were originally of the same size. He habitually uses the left in sexual intercourse.

Of course it was a matter of interest to determine whether any family history of hereditariness or any tendency to twins could be detected. In reply to interrogation on these points he stated that his father and mother were both healthy and well-formed, and he believes that there was no example of malformation of their blood relatives. His father is alive. His mother died from phthisis. He has four brothers and two sisters, all well formed and without any peculiarity of shape. His mother was not conscious of any fright during pregnancy, nor of any "maternal impression." None of his brothers or sisters are twins. He himself has always been healthy. He is very active, runs very swiftly, and is a good horseman. He usually disposes of his third limb by strapping it with webbing in the right leg of his drawers to the side and front of the right thigh. As he walks when dressed no external deformity is observable. —*London Lancet*, Jan., 1866.

---

*Literary Men and Doctors.*

It is pleasant to record the fact that nearly every literary man and woman with whom I have been acquainted, or whose lives I have looked into, has found a generous and disinterested friend in a doctor. I could, of my own knowledge, tell many anecdotes of the sacrifices made to mercy by members of the profession; of continuous labors without the thought of recompense; of anxious days and nights by sick and dying beds, without the remotest idea of "fees." I may tell one of a doctor, now himself gone home; it was related to me by Sir James Eyre, M. D. Unfortunately I have forgotten the name of the good physician, but there are, no doubt, many to whom the story will apply. Sir James called upon him one morning, when his career was but commencing, and saw his waiting-room thronged with patients. "Why," said he, "you must be getting on famously." "Well, I suppose I am," was the answer; "but let me tell this fact to *you.* This morning I have seen eight patients; six of them gave me nothing, the seventh gave me a guinea, which I have just given to the eighth." Such a Physician Providence sent to Thomas Hood.—*S. C. Hall's Biography of T. Hood.*

---

*Aneurism of the Sciatic Artery Treated by Injections of Perchlorure de Fer.*

M. Nelaton treated some time since an aneurism of the terminal portion of the sciatic artery, projecting from the buttock, by injections of the *perchlorure de fer.* The case was the most interesting as the patient had formerly had an aneurism in the same region, for which the artery had been tied above the tumor. After a single injection the pulsation had entirely disappeared, the tumor gradually diminished, no inflammation followed, and at the end of a month the patient was perfectly well. —*Gazette Medicale*, Montreal, from *Gaz. des Hopitaux.*

*Human Deterioration.*

There is a tendency perhaps in city life to diminish the size of the human form (increasing, however, the fineness of fibre and improving the *quality*), but there is no foundation for the very common belief that man has deteriorated from earlier age. The *Scottish Guardian* says: "It is a very common opinion that in the early ages of the world men in general possessed superior physical proportions, and were of a greater size than they are at present, and this notion of diminished stature and strength seems to have been just as prevalent in ancient times as at the present. Pliny observes of the human height, that 'the whole race of mankind is daily becoming smaller,'—an alarming prospect if it had been true. Homer more than once makes a very disparaging comparison between his own degenerated cotemporaries and the heroes of the Trojan war. But all the facts of the circumstances which can be brought forward on this subject tend to convince us that the human form has not degenerated, and that men of the present age are of the same stature as in the beginning of the world. In the first place, though we read both in sacred and profane history of giants, yet they were, at the time when they lived, esteemed as wonders, and far above the ordinary proportions of mankind. All the remains of the human body (as bones, and particularly the teeth) which have been found unchanged in the most ancient urns and burial-places, demonstrate this point clearly. The oldest coffin is in the great pyramid of Egypt, and M. Greaves observes that this sarcophagus hardly exceeds the size of ordinary coffins, being scarcely six feet and a half long. From looking also at the height of mummies which have been brought to this country, we must conclude that those who inhabited Egypt two or three thousand years ago were not superior in size to the present inhabitants of that country. Lastly, all the facts which we can collect from ancient works of art, from armor, as helmets and breastplates, or from buildings designed for the abode and accommodation of men, concur in strengthening the proof against the decay of nature. That man is not degenerated in stature in consequence of the effects of civilization is clear, because the inhabitants of savage countries, as the natives of America, Africa, Australia, or the South Sea Islands, do not exceed us in size."

---

*Ventilation of Sewers.*

M. Robinet, a French chemist, has devised a means of freeing the sewers from the effluvia which escape in the attempt to ventilate them. He proposes that the furnaces of factories shall derive their supply of air from the sewers. The gases will be destroyed by combustion, fresh air from the atmosphere supplying their place. He calculates that if the combustion of only 70,000 tons of coal can thus be economized annually in Paris, or only a tenth part of what is burned there, the sewers will be supplied with about 140,000,000 cubic feet of fresh air—that is, more than seven times their contents—daily. He would apply the same principle to the ventilation of cesspools, etc. It has been partially in use already, on the small scale.—*Scientific Review.*

---

*Trichinæ.*

Prof. Virchow, in his *Essay on Trichinæ*, says, that the most careful cooking is required to destroy the trichinæ in fresh meat; that salt has a very destructive effect on the parasites; and that ten days salting will effectually destroy them. Hams, sausages, etc., should not be eaten fresh; but should be kept a long time before being used.—*Brit. Med. Journ.*, July 22, 1865.

---

*Regeneration of Bone.*

In a biographical sketch of Prof. J. R. Wood, of New York, Dr. Francis states that "Dr. Wood has reproduced almost every bone in the body by treating with profound respect the periosteum. His museum contains all the experiments on the human patient that were practiced on birds and quadrupeds by Duhamel and Flourens."

---

*Lemon Juice for Cancer.*

It is said, on the authority of a Dr. Brandini, that lemon juice, or a solution of citric acid, relieves the pain of a cancer, when applied to the sore as a lotion. The discovery was made accidentally, and the value of the application was confirmed by repeated experiments.

